# Regional differences in heart failure risk in the United Kingdom are partially explained by biological aging

**DOI:** 10.3389/fpubh.2024.1381146

**Published:** 2024-06-05

**Authors:** Jason Y. Y. Wong, Batel Blechter, Erik J. Rodriquez, Joseph J. Shearer, Charles Breeze, Eliseo J. Pérez-Stable, Véronique L. Roger

**Affiliations:** ^1^Epidemiology and Community Health Branch, National Heart Lung and Blood Institute, Bethesda, MD, United States; ^2^Occupational and Environmental Epidemiology Branch, Division of Cancer Epidemiology and Genetics, National Cancer Institute, Rockville, MD, United States; ^3^Office of the Director, National Institute on Minority Health and Health Disparities, Bethesda, MD, United States

**Keywords:** heart failure, prospective cohort study, biological aging, UK biobank, allostatic load

## Abstract

**Background:**

Heart failure (HF) risk is greater in rural versus urban regions in the United States (US), potentially due to differences in healthcare coverage and access. Whether this excess risk applies to countries with universal healthcare is unclear and the underlying biological mechanisms are unknown. In the prospective United Kingdom (UK) Biobank, we investigated urban–rural regional differences in HF risk and the mechanistic role of biological aging.

**Methods:**

Multivariable Cox regression was used to estimate the hazard ratios (HRs) and 95% confidence intervals (CIs) of incident HF in relation to residential urban–rural region and a Biological Health Score (BHS) that reflects biological aging from environmental, social, or dietary stressors. We estimated the proportion of the total effect of urban–rural region on HF mediated through BHS.

**Results:**

Among 417,441 European participants, 10,332 incident HF cases were diagnosed during the follow-up. Compared to participants in large urban regions of Scotland, those in England/Wales had significantly increased HF risk (smaller urban: HR = 1.83, 95%CI: 1.64–2.03; suburban: HR = 1.77, 95%CI: 1.56–2.01; very rural: HR = 1.61, 95%CI: 1.39–1.85). Additionally, we found a dose–response relationship between increased biological aging and HF risk (HR_per 1 SD increase_ = 1.14 (95%CI: 1.12–1.17). Increased biological aging mediated a notable 6.6% (*p* < 0.001) of the total effect of urban–rural region on HF.

**Conclusion:**

Despite universal healthcare in the UK, disparities in HF risk by region were observed and may be partly explained by environmental, social, or dietary factors related to biological aging. Our study contributes to precision public health by informing potential biological targets for intervention.

## 1 Introduction

Heart failure (HF) is a complex syndrome that can manifest from any cardiovascular disease that impairs the ability of the ventricle to fill with or eject blood (1–4). In the prospective Southern Community Cohort Study (SCCS), we identified disparities in HF risk by geographic region, in which rural residents had higher risk of incident HF compared to urban residents (5). However, that study did not address the biological mechanisms underlying the association. In the US, residual confounding by healthcare coverage and access creates barriers in disentangling etiologic pathways. Understanding biological pathways could improve precision public health (6) by identifying targets for population-level interventions.

In the United Kingdom (UK), approximately 60,000 new cases of HF are diagnosed annually and the absolute lifetime risk among 30 year-old adults is 5% (4). HF is highly fatal, with an estimated survival rate of only 45.5% five years after diagnosis (7). Health care in the UK is delivered through the National Health Service (NHS), a universal single-payer healthcare system that provides relatively standardized medical care. This reduces confounding by healthcare coverage and access, which allows deeper investigation into etiologic pathways underlying regional differences.

Multi-system allostatic load scores combine various biomarkers or physiological measurements to reflect biological aging attributed to environmental, lifestyle, and social stressors (8). The recently developed Biological Health Score (BHS) integrates information from 13 physiological or biomarker measurements including cholesterol, blood pressure, insulin-like growth factor 1 (IGF-1), C-reactive protein (CRP), among others (9, 10). Higher BHS, which reflects increased biological aging, was found to be associated with increased cardiovascular disease risk and mortality, in addition to being influenced by socioeconomic inequalities (9, 10). However, the extent to which BHS varies by geographic urban–rural region and whether the pathway to HF is mediated through BHS are unknown.

To address these knowledge gaps, we leveraged data from the UK Biobank, a prospective cohort study of nearly half a million adults with extensive demographic, clinical, and biomarker data. We examined the variation in HF risk across urban–rural regions in the UK. To gain further mechanistic insight, we assessed the association between biological aging and HF risk, and estimated the proportion of the effect of region on HF risk that was causally mediated through biological aging.

## 2 Methods

### 2.1 Study design

The UK Biobank has been described (11, 12). Briefly, the target population was adults aged 40–69 years who lived ≤40 km of 22 study centers across the UK. Each study center was accessible and placed near major roads or transit links. Nearly 9.2 million people registered in the UK’s NHS were mailed invitations and 503,317 people (5.5%) visited the assessment centers in 2006–2010 (11). Volunteers were given touchscreen questionnaires, physical examinations, and provided biological samples for molecular/genetic analyses. A heat map of the residential locations of the participants was generated based on north and east grid coordinates (1 km^2^ resolution) using ArcGIS Pro (Esri, Redlands, California, United States).

The UK Biobank study was approved by the National Information Governance Board for Health and Social Care and the NHS North West Multicenter Research Ethics Committee (REC reference: 21/NW/0157, IRAS project ID: 299116). Electronic informed consent was obtained from all participants.

### 2.2 Urban–rural residential classification

In 2004, the Urban and Rural Area Classification was introduced by the Office for National Statistics (ONS) as part of the ONS Postcode Directory as a single system to define urban and rural areas of residence in the UK based on population density. These data were obtained for the participants using the GeoConvert tool provided by the UK Data Service Census Support. Information on the postcode of residence at recruitment was uploaded to GeoConvert and matched with urban–rural area classification data generated from the UK 2001 census. The urban–rural classification categories for England/Wales and Scotland are described in Supplementary Tables S1A,B, respectively. Additionally, the 18-category combined urban–rural classification for England/Wales and Scotland used in the UK Biobank is shown in Supplementary Table S1C. To improve interpretability, we collapsed the urban–rural classifications for each region to: (1) *Scotland*-large urban (reference category); (2) *Scotland*-urban; (3) *Scotland*-rural/suburban; (4) *Scotland*-very rural; (5) *England/Wales*-urban; (6) *England/Wales*-suburban; (7) *England/Wales*-rural/suburban; and (8) *England/Wales*-very rural. When conducting analyses within England, we further collapsed the classifications into: (1) Northern urban, (2) Northern rural, (3) Southern urban, and (4) Southern rural, according to previously defined boundaries (13).

### 2.3 HF diagnosis

HF was defined using in-patient hospital diagnoses coded according to the International Classification of Disease version 9 (ICD-9; 428.0 and 428.1) and version 10 (ICD-10; I50.0, I50.1, I50.9, I11.0, I13.0, and I13.2).

### 2.4 Inclusion/exclusion criteria

Among the 502,409 participants at enrollment, we excluded 372 participants with discrepancy between genetic and self-reported sex, 2,254 participants with prevalent HF, and 19,205 participants with prevalent cardiovascular disease that are on the causal pathway to HF or are strong risk factors (i.e., angina pectoris, acute myocardial infarction, subsequent ST elevation and non-ST elevation myocardial infarction and its complications, chronic/acute ischemic heart diseases, cardiomyopathy, myocarditis, intermediate coronary syndrome, coronary atherosclerosis, acute pericarditis, occlusion/stenosis of precerebral arteries, and pulmonary edema). Our analytic sample was composed of 480,578 participants. We further restricted our main statistical analyses to 447,770 participants of European ancestry with information on urban–rural classification (93.2% of analytic sample) because the vast majority of non-European participants geographically clustered in urban areas of *England/Wales* (*n* = 24,605; 5.0% of analytic sample).

### 2.5 Prospective follow-up

Follow-up time started for each eligible participant at the date of visit to the assessment center in 2006–2010 and ended at the date of first incident HF diagnosis (outcome), death (censored), or administrative censoring (i.e., September 20th, 2021, for England and Wales and October 31st, 2021, for Scotland), whichever came first.

### 2.6 Biological health score

The BHS was calculated based on 13 biomarkers as previously described (9, 10), but with some differences. The interquartile range of each biomarker was calculated among participants free of HF, major cardiovascular disease, and any cancer diagnosis at enrollment in the overall analytic sample and in the following subgroups: (1) men aged <60 years, (2) men aged ≥60 years, (3) women aged <60 years, and (4) women aged ≥60 years. Each biomarker was then dichotomized (0 = not at risk; 1 = at risk) in the overall analytic sample and in each subgroup based on their respective distributions. The “at risk” category was the lowest (1st) quartile for high-density lipoprotein cholesterol (HDL) and insulin-like growth factor 1 (IGF-1); and the highest (4th) quartile for glycated hemoglobin (HbA1c), low-density lipoprotein cholesterol (LDL), triglycerides (Tri), systolic blood pressure (SBP), diastolic blood pressure (DBP), pulse rate, C-Reactive Protein (CRP), alanine transaminase (ALT), aspartate transaminase (AST), gamma glutamyltransferase (GGT), and circulating creatinine (Cre). If the value of a biomarker was missing, a value of zero was assigned to be conservative in calculating the overall BHS. In the overall analytic sample and each subgroup, the BHS for each participant was calculated by summing the “at risk” categories of each biomarker and dividing by the number of biomarkers. To check the face validity of the BHS, we used Spearman’s rank tests to evaluate correlations with chronological age and leukocyte telomere length (LTL), a notable marker of biological aging (14).

### 2.7 Statistical analysis

#### 2.7.1 Urban–rural region and HF risk

Multivariable Cox regression was used to estimate the hazard ratios (HRs) and 95% confidence intervals (CIs) of incident hospital-diagnosed HF, in relation to urban–rural residential region at enrollment. We adjusted for age at enrollment (continuous), sex (male, female), smoking status (never, former, current), body mass index (BMI; <18.5, ≥18.5 to <25, ≥25 to <30, ≥30 to <35, and ≥35 kg/m^2^), Townsend Deprivation Index (15), and alcohol intake [never, former, current occasional, current <1 drink/day, current 1–3 drinks/day, current >3 drinks/day, unknown (16)], diabetes status (none, diabetic, unknown), glycated hemoglobin (HbA1c, mmol/mol, continuous), and hypertension status (17) using average systolic and diastolic blood pressure at baseline (normal, elevated, stage 1 and 2 hypertension, hypertensive crisis, and unknown). Follow-up time was used as the underlying timescale (18, 19).

Multiplicative effect modification was assessed using cross-product terms between urban–rural region and sex, smoking status, and age. Additionally, we conducted stratified analyses separately by sex, smoking status (never, former, current), age groups (18 to <40, ≥40 to <60, ≥60 years), and constituent country (Scotland, England, Wales). Heterogeneity was assessed using tests of contrast. In attenuation analyses, we further adjusted for time living at current residence, LTL, and BHS. We conducted additional sensitivity analyses including non-European participants. Here, changes in the effect estimates ≥10% were considered noteworthy.

Those with missing categorical covariate data were assigned a missing category in the analyses. A miniscule number of subjects were missing data on continuous Townsend Deprivation Index (*n* = 410; <1% of the analytic sample) and were excluded from the analyses.

#### 2.7.2 Biological aging and HF risk

Multivariable Cox regression models were used to estimate HRs and 95% CIs of incident HF, in relation to the per 1 SD increase in BHS values. We adjusted for study assessment center, age at enrollment, sex (in the overall analysis), smoking status, body mass index, material deprivation, diabetes, hypertension status, and alcohol intake. Additionally, we estimated the associations of HF risk with each marker separately. Here, we compared the “at risk” to the “not at risk” category for each marker. We used Z-score sign tests to assess pairwise differences in the HR estimates among subgroups.

Two-sided *p*-values <0.05 were considered statistically significant. The statistical analyses above were conducted using SAS version 9.4 (Cary, NC, United States).

#### 2.7.3 Causal mediation analyses

We evaluated whether the association between urban–rural region and HF risk was causally mediated through BHS using the Med4Way package in STATA/MP 13.0 (StataCorp LP, College Station, TX) (20). This method decomposes the total effect into four components that correspond to the proportion due to: (1) pure direct effect of exposure on outcome; (2) just interaction between the exposure and potential mediator; (3) both mediation and interaction between exposure and potential mediator; and (4) pure indirect effect through mediator (20). We simplified the categorization of urban–rural region into a binary variable with large urban areas of *Scotland* as the reference group and the other rural, rural/suburban, suburban areas of *England/Wales* as the parameter of interest. We parsimoniously adjusted for age, smoking status, sex, BMI, and alcohol intake.

## 3 Results

### 3.1 Study population

**Figure 1:**
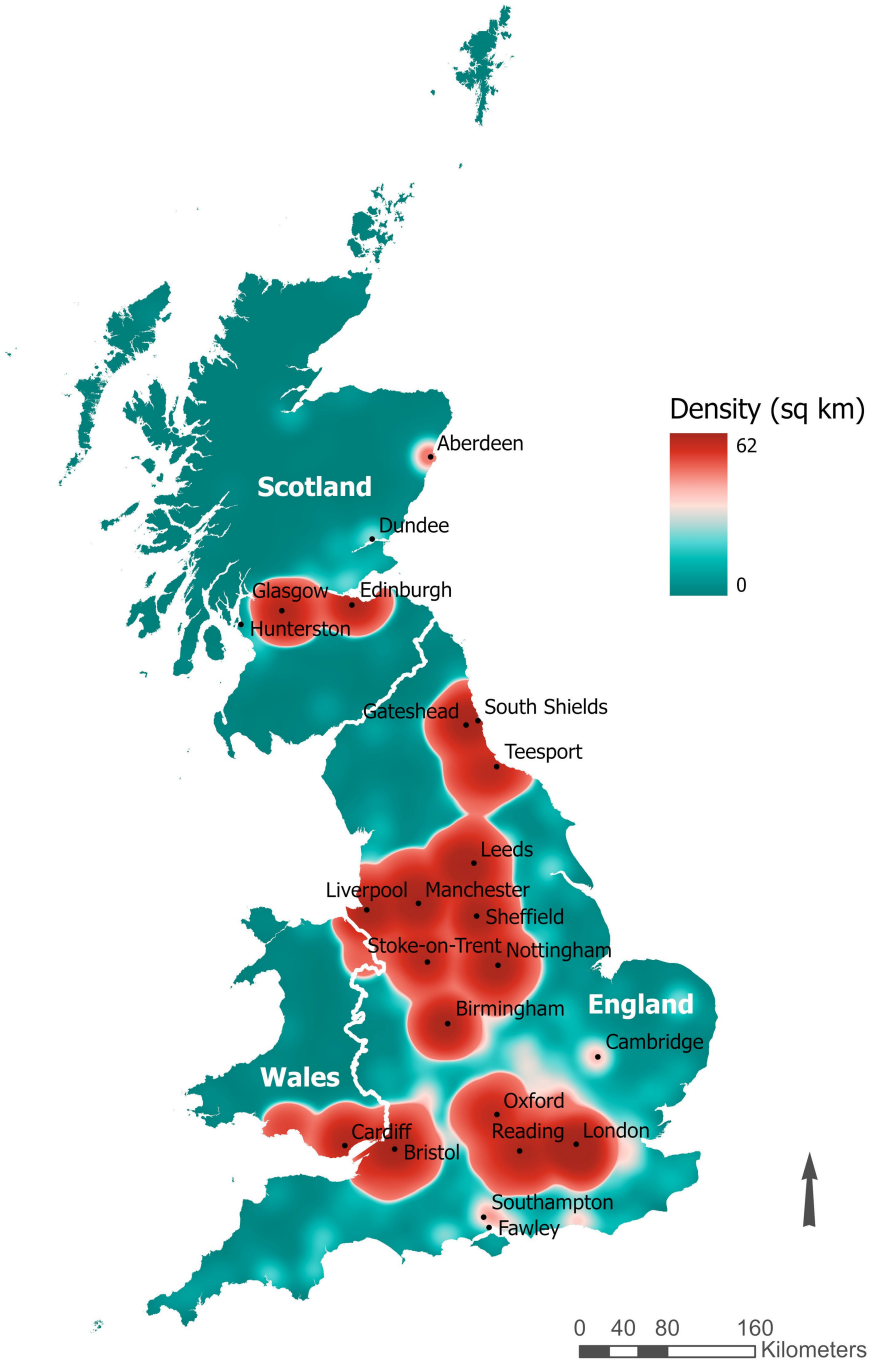
Heat map of the residential locations of the UK Biobank participants based on north and east grid coordinates (1 km^2^ resolution).

The study population characteristics are presented in Table 1. The average age at enrollment was 56.53 (8.03 SD) years. Furthermore, the study population was residentially stable, having lived an average of 17.31 (12.44 SD) years at their current residence. Among the participants, 5.7% lived in large urban areas of *Scotland*, 78.7% lived in smaller urban areas of *England/Wales*, and 15.6% lived in rural or suburban areas throughout the UK (Table 1; Figure 1).

**Table 1:** Characteristics of European study participants in the UK Biobank by geographic region and urban–rural classification (*n* = 447,770).

	I) Scotland				II) England/Wales				
	Large urban	Urban	Rural/Suburban	Very rural	Urban	Rural/Suburban	Suburban	Very rural	*p*-value
*n*	25,479	4,747	1,300	1,508	352,387	9,657	30,739	21,953	
Crude incidence rate of heart failure (per 10,000 person-years; 95% CIs)	12.9 (11.7, 14.2)	12.1 (9.5, 15.3)	17.4 (11.5, 25.1)	11.0 (6.8, 16.9)	21.3 (20.9, 21.8)	16.1 (13.9, 18.7)	19.5 (18.0, 21.0)	16.2 (14.7, 17.9)	
Baseline characteristic									
Age at enrollment, years, mean, SD	56.00	56.74	56.77	56.78	56.47	57.22	57.25	57.25	
	(8.01)	(8.03)	(7.89)	(8.1)	(8.05)	(7.72)	(7.83)	(7.74)	<0.0001
Body mass index, kg/m^2^, mean SD	27.15	27.97	28.00	27.14	27.38	26.73	27.19	26.77	
	(4.72)	(4.85)	(4.93)	(4.59)	(4.78)	(4.3)	(4.53)	(4.30)	<0.0001
Sex, *n*, %									
Women	14,593	2,629	742	847	195,345	5,396	17,221	12,309	
	(57.27)	(55.38)	(57.08)	(56.17)	(55.43)	(55.88)	(56.02)	(56.07)	<0.0001
Men	10,886	2,118	558	661	157,042	4,261	13,518	9,644	
	(42.73)	(44.62)	(42.92)	(43.83)	(44.57)	(44.12)	(43.98)	(43.93)	
Smoking status, *n*, %									
Never	14,327	2,635	717	877	189,408	5,766	17,700	12,823	
	(56.23)	(55.51)	(55.15)	(58.16)	(53.75)	(59.71)	(57.58)	(58.41)	<0.0001
Former	7,930	1,543	442	480	123,829	3,239	10,617	7,573	
	(31.12)	(32.50)	(34.00)	(31.83)	(35.14)	(33.54)	(34.54)	(34.50)	
Current	3,151	555	134	147	37,877	637	2,335	1,502	
	(12.37)	(11.69)	(10.31)	(9.75)	(10.75)	(6.60)	(7.60)	(6.84)	
Unknown	71	14	7	4	1,273	15	87	55	
	(0.28)	(0.29)	(0.54)	(0.27)	(0.36)	(0.16)	(0.28)	(0.25)	
Material deprivation, (socioeconomic status), mean, SD	−1.02	−1.37	−1.20	−2.25	−1.27	−3.12	−2.69	−3.14	
	(3.61)	(3.06)	(2.98)	(2.52)	(3.02)	(1.4)	(2.08)	(1.56)	<0.0001
Alcohol intake, *n*, %									
Never	916	168	44	47	11,506	228	847	519	
	(3.60)	(3.54)	(3.38)	(3.12)	(3.27)	(2.36)	(2.76)	(2.36)	<0.0001
Former	918	169	53	47	12,227	225	863	516	
	(3.60)	(3.56)	(4.08)	(3.12)	(3.47)	(2.33)	(2.81)	(2.35)	
Current occasional	5,524	1,214	324	319	79,382	1,626	6,206	3,758	
	(21.68)	(25.57)	(24.92)	(21.15)	(22.53)	(16.84)	(20.19)	(17.12)	
Current <1 drink/day	5,830	1,123	305	380	90,197	2,575	8,384	5,947	
	(22.88)	(23.66)	(23.46)	(25.20)	(25.60)	(26.66)	(27.27)	(27.09)	
Current 1–3 drinks/day	9,875	1,690	458	587	128,444	4,090	11,906	9,213	
	(38.76)	(35.60)	(35.23)	(38.93)	(36.45)	(42.35)	(38.73)	(41.97)	
Current >3 drinks/day	2,381	376	114	126	30,219	907	2,510	1,985	
	(9.34)	(7.92)	(8.77)	(8.36)	(8.58)	(9.39)	(8.17)	(9.04)	
Unknown	35	7	2	2	412	6	23	15	
	(0.14)	(0.15)	(0.15)	(0.13)	(0.12)	(0.06)	(0.07)	(0.07)	
Diabetes status, *n*, %									
None	24,451	4,516	1,243	1,453	335,914	9,328	29,494	21,213	
	(95.97)	(95.13)	(95.62)	(96.35)	(95.33)	(96.59)	(95.95)	(96.63)	<0.0001
Diabetic	984	227	54	54	15,623	311	1,212	700	
	(3.86)	(4.78)	(4.15)	(3.58)	(4.43)	(3.22)	(3.94)	(3.19)	
Unknown	44	4	3	1	850	18	33	40	
	(0.17)	(0.08)	(0.23)	(0.07)	(0.24)	(0.19)	(0.11)	(0.18)	
Glycated hemoglobin, HbA1c, mmol/mol, mean, SD	35.64	36.14	35.91	35.93	35.79	35.53	35.74	35.58	
	(6.36)	(6.37)	(6.71)	(6.00)	(6.32)	(5.42)	(5.94)	(5.72)	<0.0001
Hypertension status, *n*, %									
Normal	1,658	191	52	57	50,555	1,273	3,844	2,897	
	(6.51)	(4.02)	(4.00)	(3.78)	(14.35)	(13.18)	(12.51)	(13.20)	<0.0001
Elevated	1,303	187	36	55	41,263	1,170	3,511	2,570	
	(5.11)	(3.94)	(2.77)	(3.65)	(11.71)	(12.12)	(11.42)	(11.71)	
Stage 1 or 2	7,982	1,145	261	342	234,499	6,513	21,248	14,975	
	(31.33)	(24.12)	(20.08)	(22.68)	(66.55)	(67.44)	(69.12)	(68.21)	
Hypertensive crisis	284	53	9	7	7,240	227	759	545	
	(1.11)	(1.12)	(0.69)	(0.46)	(2.05)	(2.35)	(2.47)	(2.48)	
Unknown	14,252	3,171	942	1,047	18,830	474	1,377	966	
	(55.94)	(66.80)	(72.46)	(69.43)	(5.34)	(4.91)	(4.48)	(4.40)	
Biological Health Score (BHS), higher value reflects increased biological aging, mean, SD	0.214	0.230	0.221	0.211	0.239	0.227	0.241	0.229	
	(0.159)	(0.159)	(0.159)	(0.156)	(0.166)	(0.163)	(0.166)	(0.163)	<0.0001
Leukocyte telomere length, adjusted relative T/S ratio, mean, SD	0.839	0.831	0.832	0.845	0.831	0.833	0.831	0.830	
	(0.131)	(0.126)	(0.126)	(0.130)	(0.131)	(0.131)	(0.128)	(0.128)	<0.0001

Continuous variables were compared across urban–rural population density categories using Kruskal–Wallis tests, whereas categorical variables were compared using Chi-square tests. Among the 452,213 participants of European ancestry, 447,770 participants had information on urban–rural classification. Discrepancy in counts were due to missing data.

The distributions of age, BMI, material deprivation, sex, smoking status, alcohol intake, diabetes status, HbA1C, hypertension status, BHS, and LTL varied among urban–rural regions (*p*-values <0.0001; Table 1). Large urban areas in *Scotland* had the highest degree of material deprivation [−1.02 (3.61 SD)] as well as the highest proportion of current smokers (12.37%). However, those in England/Wales had markedly higher proportions of elevated blood pressure, hypertension, and biological aging compared to Scotland overall.

### 3.2 Face validity of BHS as reflective of biological aging

There was a modest positive correlation between overall BHS and chronological age (Spearman rho = 0.19, *p* < 0.0001). Additionally, we found a slight correlation between higher BHS and shorter LTL (Spearman rho = −0.08, *p* < 0.0001), as well as a slight correlation between increased material deprivation and higher BHS (Spearman rho = 0.04, *p* < 0.0001).

### 3.3 Associations between urban–rural region and HF risk

Among 417,441 European participants who were free of HF and cardiovascular diseases at enrollment, we identified 10,332 incident cases of HF during the follow-up (Mean (SD): 12.3 (1.8) years; 5.1 million person-years overall). The average age at diagnosis was 70.3 (7.0 SD) years and the average follow-up time to diagnosis was 8.3 (3.3 SD) years.

Participants who lived in less-densely populated regions of *England/Wales* had higher risk of HF compared to those who lived in large urban areas of *Scotland* (*p*-heterogeneity <0.0001; Table 2). In particular, residents of smaller urban (HR = 1.83, 95% CI: 1.64–2.03, *p* < 0.0001), suburban (HR = 1.77, 95% CI: 1.56–2.01, *p* < 0.0001), rural/suburban (HR = 1.59, 95% CI: 1.33–1.91, *p* < 0.0001), and very rural (HR = 1.61, 95% CI: 1.39–1.85, *p* < 0.0001) regions of *England/Wales* had similarly elevated HF risk (Table 2). We did not observe a monotonic exposure-response relationship between rurality and HF risk overall or separately within England and Scotland (Table 2).

**Table 2:** Urban–rural region and risk of incident heart failure among European participants in the UK Biobank.

Region/Population density	I) Overall: 10,332 cases/417,441 participants						II) Men: 6,171 cases/186,007 participants						III) Women: 4,161 cases/231,434 participants					
	No. of incident heart failure cases	Hazard ratio	95% CI Lower	95% CI Upper	*p*-value		No. of incident heart failure cases	Hazard ratio	95% CI Lower	95% CI Upper	*p*-value		No. of incident heart failure cases	Hazard ratio	95% CI Lower	95% CI Upper	*p*-value	
Scotland—Large Urban (REFERENCE)	413	1.00					240	1.00					173	1.00				
Scotland—Urban	72	0.88	0.68	1.13	0.30		44	0.91	0.66	1.25	0.55		28	0.83	0.56	1.24	0.36	
Scotland—Rural/Suburban	28	1.21	0.82	1.77	0.34		14	1.00	0.58	1.71	1.00		14	1.53	0.89	2.64	0.12	
Scotland—Very Rural	21	0.88	0.57	1.36	0.56		18	1.24	0.77	2.01	0.38		3	0.31	0.10	0.98	0.05	*
England/Wales—Urban	8,510	1.83	1.64	2.03	<0.0001	*	5,058	1.80	1.56	2.07	<0.0001	*	3,452	1.86	1.58	2.20	<0.0001	*
England/Wales—Suburban	691	1.77	1.56	2.01	<0.0001	*	431	1.82	1.54	2.15	<0.0001	*	260	1.70	1.39	2.08	<0.0001	*
England/Wales—Rural/Suburban	182	1.59	1.33	1.91	<0.0001	*	111	1.56	1.23	1.96	0.0002	*	71	1.65	1.24	2.19	0.0006	*
England/Wales—Very Rural	415	1.61	1.39	1.85	<0.0001	*	255	1.60	1.33	1.93	<0.0001	*	160	1.61	1.28	2.01	<0.0001	*

(I) Among participants of European ancestry, multivariable Cox regression models were used to estimate hazard ratios (HR) and 95% confidence intervals (CI) of incident heart failure, in relation to rural–urban classification of the participants’ residence at baseline. The models were further adjusted for potential confounders including age at enrollment (continuous), sex (men, women), smoking status (never, former, current), body mass index (BMI; <18.5, ≥18.5 to <25, ≥25 to <30, ≥30 to <35, and ≥35 kg/m^2^), material deprivation (continuous), and alcohol intake (never, former, current occasional, current < 1 drink/day, current 1–3 drinks/day, current > 3 drinks/day, unknown), diabetes status (none, diabetic, unknown), glycated hemoglobin (HbA1c, mmol/mol, continuous), and hypertension status based on American Heart Association/American College of Cardiology cutoffs using average systolic and diastolic blood pressure at baseline (normal, elevated, stage 1 and 2 hypertension, hypertensive crisis, and unknown). (II and III) Multivariable Cox regression models stratified by sex had sex removed as a covariate. *p < 0.05.

In attenuation analyses, adjustment for biological aging or LTL did not change the urban–rural effect estimates (data not shown). In sensitivity analyses, we found similar associations between urban–rural region and HF risk when stratified by sex (Table 2), smoking (Supplementary Table S2), and age groups (Supplementary Table S3). There was no evidence for multiplicative interactions of urban–rural region with sex, age, or smoking (*p*-interactions >0.05). We also included non-European participants and found similar results as the main analyses (Supplementary Table S4).

We found that participants who lived in urban areas of Northern England had a moderately higher risk of HF compared to those who lived in urban areas of Southern England (HR = 1.16, 95%CI: 1.11–1.21, *p* < 0.0001), which was consistent for men and women (Supplementary Table S5).

### 3.4 Biological aging mediates the pathway to HF

We found a dose–response relationship between increased biological aging and elevated HF risk (HR_per 1 SD increase in BHS_ = 1.14 (95% CI: 1.12–1.17, *p* < 0.0001; Table 3). The subgroup specific BHS findings were similar to the overall results (Table 3). However, the effect estimates among women aged <60 years were more pronounced compared to the overall sample (HR_per 1 SD increase in BHS_ = 1.25 (95% CI: 1.10–1.19, *p* < 0.0001; *p*-difference = 0.02). Most of the component markers used to derive the BHS were independently associated with increased HF risk (Supplementary Table S6) and there was low-to-moderate correlation among the markers (Supplementary Table S7).

**Table 3:** Biological aging from environmental and social stressors and risk of incident heart failure among Europeans.

Subgroup	No. of participants	Average biological health score	SD	No. of incident heart failure cases	Hazard ratio, per 1 SD increase	95% CI Lower	95% CI Upper	*p*-value	
Overall	451,677	0.236	0.165	10,391	1.14	1.12	1.17	<0.0001	*
Men, <60 years	112,196	0.235	0.161	1,695	1.17	1.12	1.22	<0.0001	*
Men, ≥60 years	88,454	0.237	0.150	4,517	1.12	1.09	1.16	<0.0001	*
Women, <60 years	144,651	0.236	0.171	1,011	1.25	1.16	1.34	<0.0001	*
Women, ≥60 years	106,376	0.238	0.156	3,168	1.15	1.10	1.19	<0.0001	*

Among participants of European ancestry, multivariable Cox regression models were used to estimate hazard ratios (HR) and 95% confidence intervals (CI) of incident heart failure, in relation to continuous biological health score (per 1 SD increase in the subgroup specific value). The models were further adjusted for potential confounders including age at enrollment (continuous), sex (men, women) only in the overall analyses, smoking status (never, former, current), body mass index (BMI; <18.5, ≥18.5 to <25, ≥25 to <30, ≥30 to <35, and ≥35 kg/m^2^), material deprivation (continuous), and alcohol intake (never, former, current occasional, current < 1 drink/day, current 1–3 drinks/day, current > 3 drinks/day, unknown), diabetes status (none, diabetic, unknown), and hypertension status based on American Heart Association/American College of Cardiology cutoffs 20 using average systolic and diastolic blood pressure at baseline (normal, elevated, stage 1 and 2 hypertension, hypertensive crisis, and unknown). Sex was removed as a covariate in the subgroup analyses. *p < 0.05.

When examining the distribution of biological aging by urban–rural region, we found evidence for heterogeneity (Kruskal–Wallis *p*-values <0.0001; Supplementary Table S8). Compared to large urban areas of *Scotland*, less-populated regions of *England/Wales* had slightly higher biological aging in the range of 1.2–2.6% (Supplementary Table S8).

We found that the total effect of urban–rural region on HF risk was partially mediated through increased biological aging (Table 4). The pure indirect effect through BHS accounted for 6.6% (Coefficient = 0.051, 95% CI: 0.038–0.063, *p* < 0.001) of the total effect (Coefficient = 0.768, 95% CI: 0.580–0.956, *p* < 0.001) (Table 4).

**Table 4:** Mediation analysis to decompose the total effect of urban–rural region on heart failure risk through biological aging from environmental and social stressors.

Effect	Coefficient	95% CI Lower	95% CI Upper	Proportion of total effect (%)	*p*-value	
Total excess relative risk	0.768	0.580	0.956	Ref	<0.001	*
Excess relative risk due to controlled direct effect	0.822	0.477	1.167	107.1	<0.001	*
Excess relative risk due to interaction with reference group	−0.115	−0.410	0.180	−14.9	0.45	
Excess relative risk due to mediated interaction	0.010	−0.001	0.021	1.3	0.09	
Excess relative risk due to pure indirect effect	0.051	0.038	0.063	6.6	<0.001	*

In the mediation analyses, we analyzed 10,353 incident heart failure cases among 423,521 European participants. We simplified the categorization of urban–rural classification into a binary variable with large urban areas in Scotland as the reference group and the rural or less-densely populated areas of England/Wales as the parameter of interest. We did not include the very rural or rural/suburban areas of Scotland in the mediation analyses due to sparse case numbers. We adjusted the Cox regression model for the outcome as well as the linear regression model for the mediator with a minimal set of covariates pertinent to both sub-analyses, including age, smoking status, sex, BMI, and alcohol intake. *p < 0.05.

## 4 Discussion

We investigated urban–rural regional differences in HF risk in the UK Biobank and the potential biological pathway mediating the relationship. We found that those who lived in less-densely populated areas of *England/Wales* had higher HF risk compared to participants who lived in large urban areas of *Scotland*. However, we did not observe monotonic exposure-response relationships between rurality and HF risk within the UK or its constituents. The reason for the observed lower HF risk in urban *Scotland* is unclear. Scotland has a colder climate than the rest of the UK and has limited livable land, thus most of the Scottish population is densely concentrated in Glasgow and Edinburg in the Central Lowlands (21) (Figure 1). We posit that the high population density of urban Scotland potentially reflects easier access to health services or social support/cohesiveness, thus contributing to lower HF risk.

Within England, well-documented health disparities exist between the northern and southern regions because of changing economic, political, and cultural patterns since the industrial revolution (22). Higher all-cause and cardiovascular-related mortality have been reported in Northern England (22, 23). Consistent with this historical “North–South Divide”, we found that participants who lived in urban areas of Northern England had higher HF risk compared to their Southern counterparts, as well as those from Scotland.

The biomarker composition and calculation of the BHS was first reported by Karimi et al. and Chadeau-Hyam et al. (9, 10). Similar to previous allostatic load scores, the BHS includes biomarkers reflective of biological burden to the inflammatory, cardiovascular, and metabolic systems, and also integrates biomarkers that reflect the function of two key organs, namely the liver and kidney (9, 10). As an integrative metric of biological aging or colloquially “wear-and-tear,” the BHS was previously found to track with socioeconomic status (10), and be associated with all-cause, overall cancer and CVD mortality, and CVD incidence (9) in independent cohorts. In our study, we found a strong dose–response relationship between increased biological aging and elevated HF risk that was not driven by a single outlying marker. We further investigated whether biological aging mediated the relationship between urban–rural region and HF risk and found that the indirect effect of biological aging accounted for a notable proportion of the total effect. This indirect effect was more modest than expected because although BHS was strongly associated with HF risk (Table 3), it varied slightly by urban–rural region (Supplementary Table S8). Since BHS integrates information from multiple markers and is subject to misclassification, even small detected regional differences in BHS values in a large population is noteworthy.

The biological pathway from geographic regional differences, biological aging, to HF pathogenesis is unclear and may involve multiple mechanisms upstream and downstream of biological aging. Since the BHS incorporates CRP, which is correlated with the other markers of cardiovascular, metabolic, liver, and kidney health, inflammatory dysfunction may play a central role in this pathway. CRP is an acute phase inflammatory protein synthesized primarily in liver hepatocytes and secondarily by smooth muscle cells, endothelial cells, adipocytes, and some white blood cell subtypes. CRP is often used as a clinical marker of inflammation and increased serum concentrations are consistent predictors of cardiovascular disease, including HF syndrome, in various populations (24–26). CRP is a pro-inflammatory cytokine that can trigger the release or is correlated with other inflammatory markers such as interleukin-6, interleukin-8 and tumor necrosis factor-α (27), which suggests possible involvement of specific cell-mediated immunity and apoptotic pathways as well. Interestingly, CRP has been found to reflect circulating sex hormone levels and hormone replacement therapy (24, 28–30), which we have previously linked to HF risk in the UK Biobank (31) and suggests the involvement of metabolic alterations in the pathway.

The overall findings from our study were mostly consistent with and expand upon those from our previous study conducted in the southern US (5) with important novel findings. In the SCCS, rural participants had a 19% greater overall risk of incident HF compared with urban residents (5), with associations strongest among those of African ancestry followed by White women, whereas no association was detected among White men. In the UK Biobank, we detected urban–rural regional differences in HF risk among White Europeans overall and among men and women.

Suspected factors that contribute to differences in urban–rural effects between the US and UK include healthcare systems (32, 33), dietary patterns (34), cardiovascular diseases incidence (35), geographic size and density, and societal/interpersonal factors. However, access to care has been identified as one of the most important contributors (36). In the US, decreased access to care among those living in rural areas can adversely affect future risk of HF due to inequities in preventative care and management of risk factors (33, 37). In the UK, even though the NHS reduces inequities, residents of less populated regions are still subject to challenges in geographical distances to care facilities and have differences in care-seeking behaviors, which can impact healthcare access (38).

Our study had numerous strengths. First, the prospective cohort design supported our inferences by establishing temporality between the exposure, covariates, and outcome. Second, our study had sufficient power to detect associations overall and among subgroups. Third, the UK Biobank was linked to national hospital registries; therefore, we were confident that most serious cases of HF requiring in-patient hospitalization in the cohort were captured.

Our study had some limitations. We did not have information on HF subtypes, severity, or ejection fraction. However, this misclassification was likely non-differential; therefore, the observed associations were likely underestimates of the true effect. Additionally, recruitment in the UK Biobank was achieved using a two-step process. Of the 9.2 million people mailed invitations, only 5.5% visited the assessment centers. A degree of healthy volunteer selection bias has been suggested in the UK Biobank (39, 40) and it is possible that the UK Biobank did not capture those living in inaccessible regions. Lastly, the use of national hospital registries primarily captures more serious cases of HF requiring in-patient hospitalization (fatal and non-fatal combined). Currently, preliminary primary care data, which possibly captures earlier characteristics of stage B pre-heart failure, is still being tested in the UK Biobank and is available only in a subset of participants.

Our investigation is among the few prospective cohort studies that characterized variation in HF risk by urban–rural region. We expanded on previous studies by demonstrating the role of increased biological aging in partially explaining regional variation in HF risk. Biological aging mediated a modest but notable proportion of this relationship. As such, the contribution of other mediating factors warrant further investigation. Given the differences in healthcare systems and delivery as well as other factors in Scotland compared to England/Wales, caution is recommended when interpreting the findings.

## Data availability statement

Publicly available datasets were analyzed in this study. This data can be found at: https://ams.ukbiobank.ac.uk/ams/.

## Ethics statement

The studies involving humans were approved by National Information Governance Board for Health and Social Care and the NHS North West Multicenter Research Ethics Committee (REC reference: 21/NW/0157, IRAS Project ID: 299116). The studies were conducted in accordance with the local legislation and institutional requirements. The participants provided their written informed consent to participate in this study.

## Author contributions

JW: Conceptualization, Data curation, Formal analysis, Funding acquisition, Investigation, Methodology, Project administration, Resources, Supervision, Writing – original draft, Writing – review & editing. BB: Methodology, Writing – original draft, Writing – review & editing. ER: Writing – original draft, Writing – review & editing. JS: Writing – original draft, Writing – review & editing. CB: Writing – original draft, Writing – review & editing. EP-S: Writing – original draft, Writing – review & editing. VR: Conceptualization, Writing – original draft, Writing – review & editing.
