# Supplementary material for: Regional differences in heart failure risk in the United Kingdom are partially explained by biological aging
Source: Front Public Health. 2024 Jun 5;12:1381146. doi: 10.3389/fpubh.2024.1381146 (PMC11188461; doi:10.3389/fpubh.2024.1381146)
Supplement: Supplementary file 1 [file Data_Sheet_1.DOCX]

| **Supplementary Table 1: Urban and rural area classification as defined by the Office for National Statistics (ONS)** | |
| --- | --- |
|  |  |
| **A) England and Wales** | |
| Number | Population density description |
| 1 | Urban ≥ 10k – sparse: output area falls within Urban settlements with a population of 10,000 or more and the wider surrounding area is sparsely populated; |
| 2 | Town and Fringe – sparse: output area falls within the Small Town and Fringe areas category and the wider surrounding area is sparsely populated; |
| 3 | Village – sparse: output area falls within the Village category and the wider surrounding area is sparsely populated; |
| 4 | Hamlet and Isolated Dwelling – sparse: output area falls within the Hamlet & Isolated Dwelling category and the wider surrounding area is sparsely populated; |
| 5 | Urban ≥ 10k – less sparse: output area falls within Urban settlements with a population of 10,000 or more and the wider surrounding area is less sparsely populated; |
| 6 | Town and Fringe – less sparse: output area falls within the Small Town and Fringe areas category and the wider surrounding area is less sparsely populated; |
| 7 | Village – less sparse: output area falls within the Village category and the wider surrounding area is less sparsely populated; |
| 8 | Hamlet and Isolated Dwelling – less sparse: output area falls within the Hamlet & Isolated Dwelling category and the wider surrounding area is less sparsely populated. |
| 9 | Postcode in Scotland/NI/Channel Islands/Isle of Man |
|  |  |
| **B) Scotland** | |
| Number | Population density description |
| 1 | Large Urban Area: Settlement of over 125,000 people; |
| 2 | Other Urban Area: Settlement of 10,000 to 125,000 people; |
| 3 | Accessible Small Town: Settlement of 3,000 to 10,000 people, within 30 minutes drive of a settlement of 10,000 or more; |
| 4 | Remote Small Town: Settlement of 3,000 to 10,000 people, with a drive time of 30 to 60 minutes to a settlement of 10,000 or more; |
| 5 | Very Remote Small Town: Settlement of 3,000 to 10,000 people, with a drive time of over 60 minutes to a settlement of 10,000 or more; |
| 6 | Accessible Rural: Settlement of less than 3,000 people, within 30 minutes drive of a settlement of 10,000 or more; |
| 7 | Remote Rural: Settlement of less than 3,000 people, with a drive time of 30 to 60 minutes to a settlement of 10,000 or more; |
| 8 | Very Remote Rural: Settlement of less than 3,000 people, with a drive time of over 60 minutes to a settlement of 10,000 or more |
| 9 | Postcode in England /NI/Channel Islands/Isle of Man |
|  |  |
| **C) Combined population density categories in UK Biobank** | |
| Number | Population density description |
| 1 | England/Wales - Urban - sparse |
| 2 | England/Wales - Town and Fringe - sparse |
| 3 | England/Wales - Village - sparse |
| 4 | England/Wales - Hamlet and Isolated dwelling - sparse |
| 5 | England/Wales - Urban - less sparse |
| 6 | England/Wales - Town and Fringe - less sparse |
| 7 | England/Wales - Village - less sparse |
| 8 | England/Wales - Hamlet and Isolated Dwelling - less sparse |
| 9 | Postcode not linkable |
| 11 | Scotland - Large Urban Area |
| 12 | Scotland - Other Urban Area |
| 13 | Scotland - Accessible Small Town |
| 14 | Scotland - Remote Small Town |
| 15 | Scotland - Very Remote Small Town |
| 16 | Scotland - Accessible Rural |
| 17 | Scotland - Remote Rural |
| 18 | Scotland - Very Remote Rural |

| **Supplementary Table 2: Urban-rural region and risk of incident heart failure among never, former, current smokers in the UK Biobank** | | | | | | | | | | | | | | | | | | |
| --- | --- | --- | --- | --- | --- | --- | --- | --- | --- | --- | --- | --- | --- | --- | --- | --- | --- | --- |
|  | I) Never Smokers | | | | |  | II) Former Smokers | | | | |  | III) Current Smokers | | | | | |
| Region / Population density | No. of incident heart failure cases | Hazard Ratio | 95% CI Lower | 95% CI Upper | P-value |  | No. of incident heart failure cases | Hazard Ratio | 95% CI Lower | 95% CI Upper | P-value |  | No. of incident heart failure cases | Hazard Ratio | 95% CI Lower | 95% CI Upper | P-value |  |
|  |  |  |  |  |  |  |  |  |  |  |  |  |  |  |  |  |  |  |
| Scotland - Large Urban (REFERENCE) | 162 | 1.00 |  |  |  |  | 153 | 1.00 |  |  |  |  | 97 | 1.00 |  |  |  |  |
| Scotland - Urban | 32 | 0.98 | 0.67 | 1.44 | 0.93 |  | 26 | 0.79 | 0.52 | 1.19 | 0.25 |  | 13 | 0.80 | 0.45 | 1.43 | 0.46 |  |
| Scotland - Rural/Suburban | 9 | 1.00 | 0.51 | 1.96 | 0.99 |  | 9 | 0.89 | 0.45 | 1.74 | 0.73 |  | 10 | 2.88 | 1.50 | 5.54 | 0.0015 | * |
| Scotland - Very Rural | 10 | 1.02 | 0.54 | 1.93 | 0.95 |  | 9 | 0.93 | 0.47 | 1.82 | 0.83 |  | 2 | 0.47 | 0.12 | 1.91 | 0.29 |  |
| England/Wales - Urban | 3468 | 1.97 | 1.66 | 2.33 | <0.0001 | * | 3670 | 1.83 | 1.54 | 2.18 | <0.0001 | * | 1321 | 1.57 | 1.25 | 1.97 | 0.0001 | * |
| England/Wales - Suburban | 311 | 1.93 | 1.58 | 2.36 | <0.0001 | * | 299 | 1.76 | 1.44 | 2.17 | <0.0001 | * | 76 | 1.44 | 1.05 | 1.98 | 0.02 | * |
| England/Wales - Rural/Suburban | 89 | 1.72 | 1.32 | 2.25 | <0.0001 | * | 76 | 1.61 | 1.21 | 2.14 | 0.001 | * | 17 | 1.33 | 0.78 | 2.26 | 0.29 |  |
| England/Wales - Very Rural | 180 | 1.60 | 1.28 | 2.01 | <0.0001 | * | 181 | 1.64 | 1.31 | 2.05 | <0.0001 | * | 53 | 1.73 | 1.21 | 2.46 | 0.0025 | * |
|  |  |  |  |  |  |  |  |  |  |  |  |  |  |  |  |  |  |  |
| Among participants of European ancestry, smoking-stratified multivariable Cox regression models were used to estimate hazard ratios (HR) and 95% confidence intervals (CI) of incident heart failure, in relation to rural-urban classification of the participants’ residence at baseline. The models were further adjusted for potential confounders including age at recruitment (continuous), sex (men, women), body mass index (BMI; <18.5, ≥18.5 to <25, ≥25 to <30, ≥30 to <35, and ≥35 kg/m2), material deprivation (continuous), and alcohol intake (never, former, current occasional, current <1 drink/day, current 1-3 drinks/day, current >3 drinks/day, unknown), diabetes status (none, diabetic, unknown), glycated hemoglobin (HbA1c, mmol/mol, continuous), and hypertension status based on American Heart Association/American College of Cardiology cutoffs 20 using average systolic and diastolic blood pressure at baseline (normal, elevated, stage 1 and 2 hypertension, hypertensive crisis, and unknown). *P<0.05 | | | | | | | | | | | | | | | | | | |

| **Supplementary Table 3: Urban-rural region and risk of incident heart failure by age groups in the UK Biobank** | | | | | | | | | | | | | | | | | | |
| --- | --- | --- | --- | --- | --- | --- | --- | --- | --- | --- | --- | --- | --- | --- | --- | --- | --- | --- |
|  | 1. **37 to <50 years**   Mean: 44.99 (2.74 SD years)  Median: 45 (IQR: 43-47 years) | | | | |  | 1. **≥50 to <60 years**   Mean: 54.80 (2.87 SD years)  Median: 55 (IQR: 52-57 years) | | | | |  | 1. **≥60 years**   Mean: 64.08 (2.84 SD years)  Median 64 (IQR: 62-66 years) | | | | | |
| Region / Population density | No. of incident heart failure cases | Hazard Ratio | 95% CI Lower | 95% CI Upper | P-value |  | No. of incident heart failure cases | Hazard Ratio | 95% CI Lower | 95% CI Upper | P-value |  | No. of incident heart failure cases | Hazard Ratio | 95% CI Lower | 95% CI Upper | P-value |  |
|  |  |  |  |  |  |  |  |  |  |  |  |  |  |  |  |  |  |  |
| Scotland - Large Urban (REFERENCE) | 23 | 1.00 |  |  |  |  | 112 | 1.00 |  |  |  |  | 278 | 1.00 |  |  |  |  |
| Scotland - Urban | 5 | 1.36 | 0.52 | 3.60 | 0.53 |  | 13 | 0.63 | 0.35 | 1.11 | 0.11 |  | 54 | 0.94 | 0.70 | 1.26 | 0.67 |  |
| Scotland - Rural/Suburban | 1 | 1.07 | 0.14 | 7.92 | 0.9486 |  | 9 | 1.49 | 0.75 | 2.94 | 0.25 |  | 18 | 1.12 | 0.70 | 1.81 | 0.63 |  |
| Scotland - Very Rural | 2 | 1.85 | 0.43 | 7.87 | 0.41 |  | 6 | 1.13 | 0.50 | 2.57 | 0.77 |  | 13 | 0.74 | 0.42 | 1.29 | 0.28 |  |
| England/Wales - Urban | 474 | 1.91 | 1.22 | 3.00 | 0.0048 | * | 1779 | 1.59 | 1.29 | 1.97 | <0.0001 | * | 6257 | 1.92 | 1.68 | 2.18 | <0.0001 | * |
| England/Wales - Suburban | 21 | 1.34 | 0.72 | 2.49 | 0.35 |  | 121 | 1.52 | 1.16 | 2.00 | 0.0027 | * | 549 | 1.89 | 1.62 | 2.20 | <0.0001 | * |
| England/Wales - Rural/Suburban | 9 | 2.24 | 1.01 | 4.96 | 0.0468 | * | 25 | 1.00 | 0.64 | 1.56 | 0.99 |  | 148 | 1.76 | 1.43 | 2.16 | <0.0001 | * |
| England/Wales - Very Rural | 14 | 1.41 | 0.71 | 2.83 | 0.33 |  | 70 | 1.28 | 0.93 | 1.75 | 0.13 |  | 331 | 1.73 | 1.46 | 2.04 | <0.0001 | * |
|  |  |  |  |  |  |  |  |  |  |  |  |  |  |  |  |  |  |  |
| Among participants of European ancestry, age-stratified multivariable Cox regression models were used to estimate hazard ratios (HR) and 95% confidence intervals (CI) of incident heart failure, in relation to rural-urban classification of the participants’ residence at baseline. The models were further adjusted for potential confounders including age at recruitment within each age-group (continuous), sex (men, women), smoking status (never, former, current), body mass index (BMI; <18.5, ≥18.5 to <25, ≥25 to <30, ≥30 to <35, and ≥35 kg/m^2^), material deprivation (continuous), and alcohol intake (never, former, current occasional, current <1 drink/day, current 1-3 drinks/day, current >3 drinks/day, unknown), diabetes status (none, diabetic, unknown), glycated hemoglobin (HbA1c, mmol/mol, continuous), and hypertension status based on American Heart Association/American College of Cardiology cutoffs 20 using average systolic and diastolic blood pressure at baseline (normal, elevated, stage 1 and 2 hypertension, hypertensive crisis, and unknown). *P<0.05. | | | | | | | | | | | | | | | | | | |

| **Supplementary Table 4: Urban-rural region and risk of incident heart failure in the UK Biobank including participants of European and non-European ancestry** | | | | | | |
| --- | --- | --- | --- | --- | --- | --- |
|  | 10,872 cases / 441,261 participants | | | | |  |
| Region / Population density | No. of incident heart failure cases | Hazard Ratio | 95% CI Lower | 95% CI Upper | P-value |  |
|  |  |  |  |  |  |  |
| Scotland - Large Urban (REFERENCE) | 420 | 1.00 |  |  |  |  |
| Scotland - Urban | 74 | 0.89 | 0.70 | 1.14 | 0.35 |  |
| Scotland - Rural/Suburban | 28 | 1.21 | 0.82 | 1.77 | 0.34 |  |
| Scotland - Very Rural | 21 | 0.88 | 0.56 | 1.36 | 0.55 |  |
| England/Wales - Urban | 9035 | 1.83 | 1.65 | 2.04 | <0.0001 | * |
| England/Wales - Suburban | 695 | 1.77 | 1.55 | 2.01 | <0.0001 | * |
| England/Wales - Rural/Suburban | 183 | 1.59 | 1.33 | 1.90 | <0.0001 | * |
| England/Wales - Very Rural | 416 | 1.59 | 1.38 | 1.84 | <0.0001 | * |
|  |  |  |  |  |  |  |
| Multivariable Cox regression models were used to estimate hazard ratios (HR) and 95% confidence intervals (CI) of incident heart failure, in relation to rural-urban classification of the participants’ residence at baseline. The models were further adjusted for potential confounders including age at recruitment (continuous), self-reported race/ethnicity (White European, Black/African, Mixed, South Asian, other, and unknown), sex (men, women), smoking status (never, former, current), body mass index (BMI; <18.5, ≥18.5 to <25, ≥25 to <30, ≥30 to <35, and ≥35 kg/m2), Townsend deprivation index (continuous), and alcohol intake (never, former, current occasional, current <1 drink/day, current 1-3 drinks/day, current >3 drinks/day, unknown), diabetes status (none, diabetic, unknown), glycated hemoglobin (HbA1c, mmol/mol, continuous), and hypertension status based on American Heart Association/American College of Cardiology cutoffs using average systolic and diastolic blood pressure at baseline (normal, elevated, stage 1 and 2 hypertension, hypertensive crisis, and unknown). *P<0.05 | | | | | | |

| **Supplementary Table 5: Urban-rural region and risk of incident heart failure in Northern and Southern England** | | | | | | | | | | | | | | | | | | |
| --- | --- | --- | --- | --- | --- | --- | --- | --- | --- | --- | --- | --- | --- | --- | --- | --- | --- | --- |
|  | I) Overall: 9,615 cases / 368,034 participants | | | | |  | II) Men: 5,738 cases / 164,389 participants | | | | |  | III) Women: 3,877 cases / 203,645 participants | | | | | |
| Region / Population density | No. of incident heart failure cases | Hazard Ratio | 95% CI Lower | 95% CI Upper | P-value |  | No. of incident heart failure cases | Hazard Ratio | 95% CI Lower | 95% CI Upper | P-value |  | No. of incident heart failure cases | Hazard Ratio | 95% CI Lower | 95% CI Upper | P-value |  |
|  |  |  |  |  |  |  |  |  |  |  |  |  |  |  |  |  |  |  |
| Southern England, Urban (REFERENCE) | 3725 | 1.00 |  |  |  |  | 2209 | 1.00 |  |  |  |  | 1516 | 1.00 |  |  |  |  |
| Southern England, Rural | 697 | 0.95 | 0.88 | 1.03 | 0.24 |  | 427 | 0.98 | 0.88 | 1.08 | 0.64 |  | 270 | 0.92 | 0.81 | 1.05 | 0.23 |  |
| Northern England, Urban | 4621 | 1.16 | 1.11 | 1.21 | <0.0001 |  | 2743 | 1.18 | 1.11 | 1.24 | <0.0001 |  | 1878 | 1.13 | 1.06 | 1.21 | 3E-04 |  |
| Northern England, Rural | 572 | 1.05 | 0.96 | 1.15 | 0.29 |  | 359 | 1.11 | 0.99 | 1.24 | 0.07 |  | 213 | 0.96 | 0.83 | 1.11 | 0.62 |  |
|  |  |  |  |  |  |  |  |  |  |  |  |  |  |  |  |  |  |  |
| I) Among participants of European ancestry, multivariable Cox regression models were used to estimate hazard ratios (HR) and 95% confidence intervals (CI) of incident heart failure, in relation to rural-urban classification of the participants’ residence at baseline. The models were further adjusted for potential confounders including age at recruitment (continuous), sex (men, women), smoking status (never, former, current), body mass index (BMI; <18.5, ≥18.5 to <25, ≥25 to <30, ≥30 to <35, and ≥35 kg/m2), material deprivation (Townsend deprivation index, continuous), and alcohol intake (never, former, current occasional, current <1 drink/day, current 1-3 drinks/day, current >3 drinks/day, unknown), diabetes status (none, diabetic, unknown), glycated hemoglobin (HbA1c, mmol/mol, continuous), and hypertension status based on American Heart Association/American College of Cardiology cutoffs using average systolic and diastolic blood pressure at baseline (normal, elevated, stage 1 and 2 hypertension, hypertensive crisis, and unknown). II and III) Multivariable Cox regression models stratified by sex had sex removed as a covariate. The rural category used in this analysis included suburban, rural, and very rural. *P<0.05 | | | | | | | | | | | | | | | | | | |

| **Supplementary Table 6: Associations between each component marker of the Biological Health Score and risk of heart failure by sex and age** | | | | | | | |
| --- | --- | --- | --- | --- | --- | --- | --- |
| Marker / Subgroup | No. Incident HF cases, Not at Risk Category | No. Incident HF cases, 'At Risk' Category | HR | 95% CI Lower | 95% CI Upper | P-value |  |
| Overall |  |  |  |  |  |  |  |
| HbA1C | 6824 | 4331 | 1.17 | 1.12 | 1.22 | 5E-13 |  |
| HDL | 6098 | 5057 | 1.10 | 1.05 | 1.14 | 1E-05 |  |
| LDL | 8966 | 2189 | 0.84 | 0.80 | 0.88 | 3E-13 |  |
| Triglycerides | 7842 | 3313 | 0.98 | 0.94 | 1.02 | 3E-01 |  |
| SBP | 7114 | 4041 | 1.15 | 1.10 | 1.20 | 2E-09 |  |
| DBP | 8072 | 3083 | 1.04 | 0.99 | 1.09 | 1E-01 |  |
| Pulse Rate | 10076 | 1079 | 1.24 | 1.15 | 1.33 | 4E-09 |  |
| CRP | 6885 | 4270 | 1.47 | 1.41 | 1.53 | <1E-28 |  |
| IGF-1 | 6702 | 4453 | 1.08 | 1.04 | 1.13 | 7E-05 |  |
| ALT | 8255 | 2900 | 0.94 | 0.90 | 0.98 | 7E-03 |  |
| AST | 7956 | 3199 | 1.09 | 1.05 | 1.14 | 3E-05 |  |
| GGT | 7336 | 3819 | 1.27 | 1.22 | 1.32 | <1E-28 |  |
| Creatinine | 7573 | 3582 | 1.12 | 1.08 | 1.18 | 2E-07 |  |
| Men <60 years |  |  |  |  |  |  |  |
| HbA1C | 1105 | 714 | 1.57 | 1.41 | 1.74 | 1E-16 |  |
| HDL | 1049 | 770 | 1.05 | 0.95 | 1.16 | 3E-01 |  |
| LDL | 1418 | 401 | 0.96 | 0.86 | 1.08 | 5E-01 |  |
| Triglycerides | 1329 | 490 | 0.96 | 0.86 | 1.06 | 4E-01 |  |
| SBP | 1227 | 592 | 1.42 | 1.27 | 1.59 | 5E-10 |  |
| DBP | 1295 | 524 | 1.08 | 0.97 | 1.22 | 2E-01 |  |
| Pulse Rate | 1642 | 177 | 1.28 | 1.07 | 1.54 | 7E-03 |  |
| CRP | 1109 | 710 | 1.53 | 1.39 | 1.70 | 9E-17 |  |
| IGF-1 | 1076 | 743 | 1.30 | 1.18 | 1.44 | 9E-08 |  |
| ALT | 1362 | 457 | 0.85 | 0.76 | 0.95 | 4E-03 |  |
| AST | 1348 | 471 | 1.02 | 0.92 | 1.13 | 7E-01 |  |
| GGT | 1251 | 568 | 1.18 | 1.06 | 1.31 | 2E-03 |  |
| Creatinine | 1410 | 409 | 1.07 | 0.95 | 1.19 | 3E-01 |  |
| Men >=60 years |  |  |  |  |  |  |  |
| HbA1C | 1332 | 487 | 1.54 | 1.36 | 1.75 | 2E-11 |  |
| HDL | 2780 | 2028 | 1.10 | 1.03 | 1.16 | 3E-03 |  |
| LDL | 3980 | 828 | 0.75 | 0.70 | 0.81 | 3E-13 |  |
| Triglycerides | 3601 | 1207 | 0.93 | 0.87 | 0.99 | 2E-02 |  |
| SBP | 3503 | 1305 | 1.20 | 1.12 | 1.29 | 5E-07 |  |
| DBP | 3642 | 1166 | 0.96 | 0.89 | 1.03 | 2E-01 |  |
| Pulse Rate | 4325 | 483 | 1.23 | 1.10 | 1.36 | 2E-04 |  |
| CRP | 3139 | 1669 | 1.49 | 1.40 | 1.58 | <1E-28 |  |
| IGF-1 | 3140 | 1668 | 1.10 | 1.03 | 1.16 | 4E-03 |  |
| ALT | 3679 | 1129 | 0.84 | 0.78 | 0.90 | 4E-07 |  |
| AST | 3584 | 1224 | 1.05 | 0.98 | 1.12 | 2E-01 |  |
| GGT | 3382 | 1426 | 1.24 | 1.17 | 1.33 | 4E-11 |  |
| Creatinine | 3441 | 1367 | 1.27 | 1.20 | 1.36 | 7E-14 |  |
| Women <60 years |  |  |  |  |  |  |  |
| HbA1C | 678 | 417 | 1.35 | 1.18 | 1.54 | 1E-05 |  |
| HDL | 571 | 524 | 1.04 | 0.91 | 1.18 | 6E-01 |  |
| LDL | 815 | 280 | 1.00 | 0.87 | 1.15 | 1E+00 |  |
| Triglycerides | 696 | 399 | 1.17 | 1.02 | 1.33 | 2E-02 |  |
| SBP | 708 | 387 | 1.42 | 1.23 | 1.65 | 3E-06 |  |
| DBP | 744 | 351 | 1.05 | 0.91 | 1.22 | 5E-01 |  |
| Pulse Rate | 972 | 123 | 1.36 | 1.09 | 1.69 | 6E-03 |  |
| CRP | 595 | 500 | 1.69 | 1.47 | 1.93 | 7E-14 |  |
| IGF-1 | 598 | 497 | 1.39 | 1.23 | 1.57 | 2E-07 |  |
| ALT | 743 | 352 | 1.11 | 0.97 | 1.27 | 1E-01 |  |
| AST | 759 | 336 | 1.32 | 1.16 | 1.50 | 3E-05 |  |
| GGT | 663 | 432 | 1.49 | 1.31 | 1.69 | 2E-09 |  |
| Creatinine | 804 | 291 | 1.23 | 1.07 | 1.41 | 3E-03 |  |
| Women >=60 years |  |  |  |  |  |  |  |
| HbA1C | 824 | 271 | 1.49 | 1.27 | 1.75 | 1E-06 |  |
| HDL | 1879 | 1554 | 1.11 | 1.03 | 1.19 | 5E-03 |  |
| LDL | 2794 | 639 | 0.78 | 0.72 | 0.86 | 4E-08 |  |
| Triglycerides | 2483 | 950 | 0.97 | 0.90 | 1.05 | 4E-01 |  |
| SBP | 2425 | 1008 | 1.31 | 1.20 | 1.42 | 5E-10 |  |
| DBP | 2535 | 898 | 0.96 | 0.88 | 1.05 | 4E-01 |  |
| Pulse Rate | 3100 | 333 | 1.04 | 0.92 | 1.18 | 5E-01 |  |
| CRP | 2165 | 1268 | 1.43 | 1.33 | 1.55 | 4E-21 |  |
| IGF-1 | 2162 | 1271 | 1.13 | 1.06 | 1.22 | 5E-04 |  |
| ALT | 2576 | 857 | 0.90 | 0.83 | 0.98 | 1E-02 |  |
| AST | 2554 | 879 | 1.09 | 1.01 | 1.18 | 3E-02 |  |
| GGT | 2310 | 1123 | 1.31 | 1.22 | 1.42 | 4E-13 |  |
| Creatinine | 2442 | 991 | 1.25 | 1.16 | 1.35 | 5E-09 |  |
| The interquartile range of each biomarker was calculated among participants free of heart failure, major cardiovascular disease, and any cancer diagnosis at baseline in the overall analytic sample and in the following subgroups: 1) men aged <60 years, 2) men aged ≥60 years, 3) women aged <60 years, and 4) women aged ≥60 years. Each biomarker was then dichotomized (0=not at risk; 1=at risk) in the overall analytic sample and in each subgroup based on their respective distributions. The ‘at risk’ category was the lowest (1st) quartile for high-density lipoprotein cholesterol (HDL) and insulin-like growth factor 1 (IGF-1); and the highest (4th) quartile for glycated hemoglobin (HbA1c; mmol/mol), low-density lipoprotein cholesterol (LDL; mmol/l), triglycerides (mmol/l), systolic blood pressure (SBP; mmHg), diastolic blood pressure (DBP; mmHg), pulse rate (bpm), C-Reactive Protein (CRP; mg/L), alanine transaminase (ALT; U/L), aspartate transaminase (AST; U/L.), gamma glutamyltransferase (GGT; U/L), and circulating Creatinine (mmol/l). If the value of a biomarker was missing, a value of zero was assigned to be conservative in calculating the overall BHS. Among European participants, multivariable Cox regression models were used to estimate Hazard Ratios (HRs) and 95% Confidence Intervals (CIs) of incident heart failure in relation to each biomarker overall and among subgroups. The models were further adjusted for study assessment center, age at recruitment, sex (only in the overall analysis), smoking status, body mass index, material deprivation, alcohol intake, diabetes status, and hypertension status. | | | | | | | |

| **Supplementary Table 7: Spearman correlations among the 13 markers used to derive the Biological Health Score in the overall sample** | | | | | | | | | | | | | |
| --- | --- | --- | --- | --- | --- | --- | --- | --- | --- | --- | --- | --- | --- |
|  | HbA1C | HDL | LDL | Triglycerides | SBP | DBP | Pulse rate | CRP | IGF-1 | ALT | AST | GGT | Creatinine |
| HbA1C | 1 | -0.11 | 0.04 | 0.20 | 0.14 | 0.06 | 0.10 | 0.20 | -0.10 | 0.14 | 0.09 | 0.16 | 0.03 |
|  |  |  |  |  |  |  |  |  |  |  |  |  |  |
| HDL |  | 1 | 0.07 | -0.50 | -0.06 | -0.12 | -0.07 | -0.22 | -0.04 | -0.29 | -0.09 | -0.23 | -0.30 |
|  |  |  |  |  |  |  |  |  |  |  |  |  |  |
| LDL |  |  | 1 | 0.30 | 0.11 | 0.14 | 0.04 | 0.09 | -0.02 | 0.07 | 0.04 | 0.11 | -0.03 |
|  |  |  |  |  |  |  |  |  |  |  |  |  |  |
| Triglycerides |  |  |  | 1 | 0.20 | 0.20 | 0.17 | 0.26 | -0.04 | 0.34 | 0.17 | 0.36 | 0.16 |
|  |  |  |  |  |  |  |  |  |  |  |  |  |  |
| SBP |  |  |  |  | 1 | 0.70 | 0.06 | 0.16 | -0.09 | 0.20 | 0.17 | 0.25 | 0.10 |
|  |  |  |  |  |  |  |  |  |  |  |  |  |  |
| DBP |  |  |  |  |  | 1 | 0.21 | 0.17 | -0.02 | 0.23 | 0.15 | 0.26 | 0.12 |
|  |  |  |  |  |  |  |  |  |  |  |  |  |  |
| Pulse rate |  |  |  |  |  |  | 1 | 0.18 | -0.02 | 0.07 | -0.02 | 0.10 | -0.09 |
|  |  |  |  |  |  |  |  |  |  |  |  |  |  |
| CRP |  |  |  |  |  |  |  | 1 | -0.24 | 0.17 | 0.07 | 0.29 | 0.01 |
|  |  |  |  |  |  |  |  |  |  |  |  |  |  |
| IGF-1 |  |  |  |  |  |  |  |  | 1 | -0.02 | -0.11 | -0.06 | 0.10 |
|  |  |  |  |  |  |  |  |  |  |  |  |  |  |
| ALT |  |  |  |  |  |  |  |  |  | 1 | 0.70 | 0.60 | 0.22 |
|  |  |  |  |  |  |  |  |  |  |  |  |  |  |
| AST |  |  |  |  |  |  |  |  |  |  | 1 | 0.43 | 0.19 |
|  |  |  |  |  |  |  |  |  |  |  |  |  |  |
| GGT |  |  |  |  |  |  |  |  |  |  |  | 1 | 0.22 |
|  |  |  |  |  |  |  |  |  |  |  |  |  |  |
| Creatinine |  |  |  |  |  |  |  |  |  |  |  |  | 1 |
| Abbreviations: high-density lipoprotein cholesterol (HDL), insulin-like growth factor 1 (IGF-1), glycated hemoglobin (HbA1c), low-density lipoprotein cholesterol (LDL), systolic blood pressure (SBP), diastolic blood pressure (DBP), pulse rate, C-Reactive Protein (CRP), alanine transaminase (ALT), aspartate transaminase (AST), and gamma glutamyltransferase (GGT). Continuous levels of each marker were analyzed. | | | | | | | | | | | | | |

| **Supplementary Table 8: Distribution of Biological Health Scores by urban-rural region in the United Kingdom** | | | | | | | | | | | | | | | | | | | | | | | | | |
| --- | --- | --- | --- | --- | --- | --- | --- | --- | --- | --- | --- | --- | --- | --- | --- | --- | --- | --- | --- | --- | --- | --- | --- | --- | --- |
|  |  | Overall |  |  |  |  | Men, <60 years | |  |  |  | Men, ≥60 years | |  |  |  | Women, <60 years | |  |  |  | Women, ≥60 years | |  |  |
| Urban-rural classification |  | n | Average BHS | SD | ∆, % |  | n | Average BHS | SD | ∆, % |  | n | Average BHS | SD | ∆, % |  | n | Average BHS | SD | ∆, % |  | n | Average BHS | SD | ∆, % |
| Scotland - Large Urban (REFERENCE) |  | 25,467 | 0.214 | 0.159 | - |  | 6,543 | 0.210 | 0.150 | - |  | 4,337 | 0.225 | 0.149 | - |  | 9,005 | 0.212 | 0.163 | - |  | 5,582 | 0.225 | 0.154 | - |
| Scotland - Urban |  | 4,737 | 0.230 | 0.159 | 1.5 |  | 1,215 | 0.227 | 0.156 | 1.7 |  | 900 | 0.232 | 0.139 | 0.7 |  | 1,467 | 0.232 | 0.170 | 2.0 |  | 1,155 | 0.226 | 0.149 | 0.1 |
| Scotland - Rural/Suburban |  | 1,300 | 0.221 | 0.159 | 0.7 |  | 322 | 0.215 | 0.157 | 0.5 |  | 236 | 0.222 | 0.147 | -0.3 |  | 432 | 0.235 | 0.178 | 2.4 |  | 310 | 0.221 | 0.143 | -0.4 |
| Scotland - Very Rural |  | 1,505 | 0.211 | 0.156 | -0.4 |  | 354 | 0.226 | 0.157 | 1.5 |  | 306 | 0.204 | 0.136 | -2.0 |  | 493 | 0.200 | 0.162 | -1.2 |  | 352 | 0.217 | 0.142 | -0.8 |
| England/Wales - Urban |  | 352,079 | 0.239 | 0.166 | 2.4 |  | 88,201 | 0.238 | 0.162 | 2.8 |  | 68,705 | 0.239 | 0.151 | 1.4 |  | 112,580 | 0.239 | 0.172 | 2.7 |  | 82,593 | 0.240 | 0.156 | 1.5 |
| England/Wales - Suburban |  | 30,703 | 0.241 | 0.166 | 2.6 |  | 6,912 | 0.237 | 0.159 | 2.6 |  | 6,593 | 0.241 | 0.151 | 1.6 |  | 9,321 | 0.239 | 0.173 | 2.7 |  | 7,877 | 0.241 | 0.158 | 1.6 |
| England/Wales - Rural/Suburban |  | 9,644 | 0.227 | 0.163 | 1.2 |  | 2,219 | 0.225 | 0.158 | 1.5 |  | 2,037 | 0.227 | 0.147 | 0.2 |  | 2,978 | 0.220 | 0.162 | 0.9 |  | 2,410 | 0.225 | 0.151 | 0.1 |
| England/Wales - Very Rural |  | 21,925 | 0.229 | 0.163 | 1.5 |  | 4,887 | 0.228 | 0.158 | 1.7 |  | 4,741 | 0.229 | 0.147 | 0.4 |  | 6,826 | 0.227 | 0.165 | 1.5 |  | 5,471 | 0.231 | 0.154 | 0.7 |
| Kruskal-Wallis Test for Overall Heterogeneity, p-value |  | <0.0001 | | | |  | <0.0001 | | | |  | <0.0001 | | | |  | <0.0001 | | | |  | <0.0001 | | | |
